# Saint Louis University Primary Care Age-Friendly Health System Initiative in Rural and Urban Communities

**DOI:** 10.1093/geroni/igaa057.2589

**Published:** 2020-12-16

**Authors:** Marla Berg-Weger, John Morley

**Affiliations:** 1 Saint Louis University, St. Louis, Missouri, United States; 2 Saint Louis University School of Medicine, Saint Louis, Missouri, United States

## Abstract

The Saint Louis University GWEP partners with a rural Missouri critical access hospital and an urban Federally Qualified Health Center to support their transformations into an Age-Friendly Health System in their community. With an emphasis on the 4Ms, programmatic innovations include development and training of: 1) electronic health records integration of the Rapid Geriatric Assessment (RGA) for patients 65+ years old; 2) RGA-based protocol for Medicare Annual Wellness Visits (MAWV); 3) Interprofessional health care team approach; 4) Evidence-based or Evidence-Informed treatment interventions, including Cognitive Stimulation Therapy (CST), exercise and strengthening program, caregiver support, and Circle of Friends, an intervention for loneliness and social isolation. Outcomes will be presented which suggest increased assessment practices and improvement in functional and cognitive status. Successes and lessons learned regarding strategies to develop an Age-Friendly Health System in two different primary care settings will be discussed.

